# Effects of Microencapsulated Essential Oils on Growth and Intestinal Health in Weaned Piglets

**DOI:** 10.3390/ani14182705

**Published:** 2024-09-18

**Authors:** Ketian Chen, Zhiqi Dai, Yijian Zhang, Sheng Wu, Le Liu, Kai Wang, Dan Shen, Chunmei Li

**Affiliations:** 1 Research Center for Livestock Environmental Control and Smart Production, College of Animal Science and Technology, Nanjing Agriculture University, Nanjing 210000, China; ketianchen@stu.njau.edu.cn (K.C.); 2022805114@stu.njau.edu.cn (Z.D.); lle@stu.njau.edu.cn (L.L.); phd.kaiwang@pm.me (K.W.); t2022067@njau.edu.cn (D.S.); 2 Shanghai Menon Animal Nutrition Technology Co., Ltd., Shanghai 201800, China; zhangyijian1105@163.com (Y.Z.); swu_tech@sinomenon.com (S.W.)

**Keywords:** weaned piglets, microencapsulation, gut morphometry, microbiota

## Abstract

**Simple Summary:**

Weaning is a critical period in the development of piglets. During the weaning period, piglets are prone to weight loss, diarrhea, and even death due to factors such as the environment, feeding management, feed type, and feeding method. Current research indicates that essential oils can positively impact animal nutrition and production due to their antimicrobial and antioxidant properties. However, essential oils are quickly metabolized in the upper gastro-intestinal tract, so that the concentrations remaining in the distal small intestine are far too low to improve the intestinal tract. Microencapsulation technology is a relatively new processing method for essential oils. However, there is a scarcity of studies investigating the effects of microencapsulated essential oils (MEO) on growth and intestinal structure in weaned piglets. Consequently, further research on the application of MEO in the diet of weaned piglets is warranted. In this study, we administered varying concentrations of MEO to assess its impact on ADG, ADFI, FCR, diarrhea score, intestinal structure, antioxidant capacity, and other factors in weaned piglets. The aim was to develop new feed additives to enhance intestinal health of weaned piglets.

**Abstract:**

The study investigated the effects of microencapsulated essential oils (MEO) on the growth performance, diarrhea, and intestinal microenvironment of weaned piglets. The 120 thirty-day-old weaned piglets (Duroc × Landrace × Yorkshire, 8.15 ± 0.07 kg) were randomly divided into four groups and were fed with a basal diet (CON) or CON diet containing 300 (L-MEO), 500 (M-MEO), and 700 (H-MEO) mg/kg MEO, respectively, and data related to performance were measured. The results revealed that MEO supplementation increased the ADG and ADFI in weaned piglets (*p* < 0.05) compared with CON, and reduced diarrhea rates in nursery pigs (*p* < 0.05). MEO supplementation significantly increased the duodenum’s V:C ratio and the jejunal villi height of weaned piglets (*p* < 0.05). The addition of MEO significantly increased the T-AOC activity in the jejunum of piglets (*p* < 0.05), but only L-MEO decreased the MDA concentration (*p* < 0.01). H-MEO group significantly increases the content of isobutyric acid (*p* < 0.05) in the piglet colon, but it does not affect the content of other acids. In addition, MEO supplementation improved appetite in the nursery and increased the diversity and abundance of beneficial bacteria in the intestinal microbiome. In conclusion, these findings indicated that MEO supplementation improves growth and intestinal health in weaned piglets.

## 1. Introduction

From 2 to 4 weeks of age, the weaning stage is the most critical period of pig growth, during which piglets also need to cope with nutritional and environmental stress [1]. Due to the immaturity of the immune system of weaned piglets, these stressors frequently result in digestive and immune system malfunctions in piglets, leading to disorders in intestinal flora, reduced feed intake, slow growth, diarrhea, and even mortality, causing significant economic losses in swine production [2,3]. In recent years, natural nutritional supplements have been shown to effectively prevent weaning stress [4,5]. Essential oils (EOs) are natural and harmless green feed additives extracted from plants with antibacterial, antioxidant, and immune-enhancing abilities, exhibiting low toxicity, residue, and pollution [6,7,8,9,10]. Among essential oils, cinnamaldehyde, thymol, and carvacrol have been extensively researched and utilized. Cinnamaldehyde, a hydrophobic aromatic aldehyde derived from cinnamon trees, has demonstrated high antibacterial activity and is considered an effective antibiotic alternative in livestock production [11,12]. Studies have indicated that cinnamaldehyde can impede the growth of *Escherichia coli* by inducing modifications in intracellular biomacromolecules [13]. Additionally, cinnamaldehyde has been found to enhance intestinal function by augmenting the abundance of probiotics at both the phylum (*Bacteroidota* and *Firmicutes*) and genus levels (*Bacteroides* and *Lactobacillus*) [14]. Thymol is a natural monoterpenoid phenol present in various botanical families such as Lamiaceae within the thymus and origanum genera. It has a variety of biological applications, mainly focusing on expectorant, antibacterial, antifungal, antiviral, antioxidant, and anti-inflammatory activities [15,16]. Together with carvacrol, thymol is the main constituent of thyme volatile oil. Previous studies have reported that supplementation of carvacrol and thymol enhanced growth and improved the quality of meat and eggs by modulating metabolism and exhibiting antioxidative, anti-inflammatory, and antimicrobial effects in poultry production [17]. Furthermore, carvacrol and cinnamaldehyde stimulate appetite, alleviate health challenges, and reduce stress [18,19]. Additionally, supplementation of cinnamaldehyde and carvacrol somewhat improved appetite in nursery pigs, while also enhancing the diversity of the gut microbiome and increasing the abundance of beneficial bacteria [20].

Given the positive effects of cinnamaldehyde, carvacrol, and thymol in metabolic modulation, antioxidation, anti-inflammation, and antimicrobial activity, the potential synergistic benefits of combining these compounds to enhance animal growth have not been investigated. Microencapsulation technology is a rapidly developing and widely used packaging protection process. One of the advantages of microencapsulated essential oils is the precise control and sustained release of active ingredients [21]. This controlled release mechanism can target specific times or sites, facilitate penetration or wall-breaking diffusion, respond to temperature, solubility, pH fluctuations, or even the biodegradation of the capsule wall to activate release, optimizing the biological [22] and physical properties of essential oils and preventing interactions with other compounds [23,24]. Despite these benefits, there is limited research on the impact of microencapsulation technology on the growth, diarrhea rates, and intestinal health of weaned piglets. Therefore, this study aims to investigate the effects of thymol, carvacrol, and cinnamaldehyde in preparing microencapsulated essential oils (MEO) and administering various concentrations of MEO to assess their impact on weaned piglet growth performance, diarrhea rates, and intestinal health. 

## 2. Materials and Methods

Our experimental procedures used in the animal experiment were performed according to the National Institute of Health guidelines. The Animal Welfare Committee of Nanjing Agricultural University approved them (Certification No. SYXK (Su) 2022−0031). The MEO used in this experiment were provided by Shanghai Meinong (Shanghai, China) Biotechnology Co., Ltd., Shanghai, China. The product components were cinnamaldehyde 20%, thymol 2%, and carvacrol 3%; the other components were carriers.

### 2.1. Micro-Encapsulated Essential Oils

MEO preparation: stearic acid, along with other wall materials (75.0%), and a blend of essential oils (20.0% cinnamaldehyde, 2.0% thymol, 3.0% carvacrol) were heated and combined until a paste-like consistency was achieved. This mixture was then poured into a condensation spray tower and atomized into droplets, which rapidly solidified into microcapsule essential oil. Subsequently, composite mineral materials and emulsifiers were incorporated to further mix the formulation until a transparent protective film formed over the surface of the microcapsules, resulting in a double-layer microcapsule coating of essential oils.

### 2.2. Experimental Design

A total of 120 piglets (Duroc × Landrace × Yorkshire, 8.15 ± 0.07 kg) weaned at 30 d of age were randomly assigned to 4 groups (3 replicate pens per group, 10 piglets per pen), with a sex ratio of 1:1. The piglets, at 28 days of age, were pre-fed for a duration of three days, followed by a formal testing period of 14 days. They were fed a basal diet (CON) or a basal diet supplemented with 300 mg/kg MEO (L-MEO), 500 mg/kg MEO (M-MEO), and 700 mg/kg MEO (H-MEO).

Before the commencement of the experiment, the pig housing and feeding equipment were thoroughly disinfected and immunized in accordance with the standard protocols of the pig farm. Throughout the feeding period, temperature and humidity were automatically regulated (≥27 °C, ≥55%), and the pigs had ad libitum access to feed and water. The remaining materials in the feed trough were returned and weighed at 7:30 every morning and then put back into the feed trough and replenished, and the amount of each feeding was counted at 5:00 p.m. After each feeding, the feces of each pig pen were cleaned and disinfected regularly to keep the pig house clean and hygienic. The basal diet was formulated according to NRC (2012), and its ingredients and nutrient levels were shown in Table 1.

The energy level in the feed was determined using the combustion method, and the net energy was calculated accordingly. The crude protein content was assessed by the Kjeldahl method employing a Kjeldahl™ 8400( FOSS, Copenhagen, Denmark). Crude fat content was analyzed through Soxhlet extraction with an automatic fat analyzer Soxtec™ 2050 (FOSS, Copenhagen, Denmark)). The crude ash content was determined by the burning weighing method, using a muffle furnace at 550 °C for 8 h before weighing. Additionally, the total calcium content was measured using ethylenediaminetetraacetic acid complexometric titration, while the total phosphorus content was quantified by vanadium molybdenum yellow colorimetry with a spectrophotometer.

### 2.3. Sample Collection

At the end of the experiment, one piglet was randomly selected from each pen for a 24 h fast. After the fasting period, blood samples were collected through the anterior vena cava using a 5 mL disposable plastic syringe. The collected blood was then placed immediately into a vacuum blood collection tube containing a coagulant and stored in an ice box. Once the blood had coagulated, it was centrifuged at 3500 rpm for 15 min at 4 °C to extract the serum. Finally, the serum was stored at −20 °C until analysis. Following blood collection, the pigs were anesthetized with carbon dioxide and then humanely slaughtered. The heart, liver, spleen, kidney, pancreas, and lung of the fasted slaughtered piglets were taken and weighed. The small intestine was removed from the abdominal cavity and divided into three segments: duodenum, jejunum, and ileum based on their physiological characteristics, with each segment’s length measured individually. Subsequently, 2 cm of the duodenum, jejunum, and ileum were collected, washed with saline, and preserved in 4% paraformaldehyde for 24 h for morphological analysis. Additionally, certain intestinal tissues, including those from the duodenum, jejunum, and ileum, were stored at −80 °C for subsequent evaluation of their antioxidant capacity. The contents of the cecum were collected, frozen, and stored at −80 °C until microbial composition analysis was performed.

### 2.4. Growth Performance, Diarrhea, and Organ Index

Body weight was measured at the beginning and end of the experiment, and feed intake was recorded daily to calculate ADG, average daily feed intake (ADFI), and feed conversion ratio (FCR). The diarrhea status of piglets was observed at 07:00 every day on an individual pig basis (judging by whether there were wet unformed feces on the anus and hind legs). Organ indices were calculated as the percentage of body weight. This is 1 of diarrhea incidence [25]:

(1) Diarrhea incidence(%)=(the number of diarrhea pigs × diarrhea days)/(the total number of pigs × experiment days) × 100%.

### 2.5. Intestinal Tissue Morphology

In the laboratory, small intestine samples preserved in 4% paraformaldehyde were processed to prepare paraffin sections, which were subsequently stained with hematoxylin-eosin staining (H&E). Five slices were cut from each sample to measure the villus height and crypt depth, with each paraffin-embedded intestinal section having a thickness of 5 μm. Upon microscopic examination, five representative fields of view were selected from each slide to assess the intestinal mucosal morphology at low magnification (10×). ImageJ 1.8 software was utilized for measuring villus height, crypt depth, and V:C ratio.

### 2.6. Analysis of Antioxidant Capacity in Small Intestine

The total antioxidant capacity (T-AOC), malondialdehyde (MDA) content, total superoxide dismutase (T-SOD), and catalase (CAT) enzyme activities in the duodenum, jejunum, and ileum were determined using Spectrophotometric Kits (Jiancheng Bioengineering Institute, Nanjing, China) according to the manufacturer’s instructions.

### 2.7. Determination of Short-Chain Fatty Acids

We put 0.3 g of colonic chyme into a 2 mL centrifuge tube. We added 1 mL of ultrapure water, shook it thoroughly, and mixed well. We centrifuged the mixture at 13,000× *g* for 30 min. After centrifugation, we carefully aspirated 500 μL of the supernatant and added 100 μL of a 25% (*w*/*v*) crotonic acid metaphosphate solution. We incubated the mixture overnight at −20 °C. Following incubation, we centrifuged it at 10,000× *g* for 10 min, aspirated the supernatant, and filtered it with a 0.22 μm filter membrane before measurement.

Detection was performed using an Agilent GC-14B gas chromatograph (Shimadzu, Japan) with a NUKOL™ Capillary Column (Supelco; column No.34292−07B). The gas chromatograph parameters were configured as follows: the chromatographic column was operated at a column temperature of 130 °C, the vaporization temperature was set to 180 °C, and a hydrogen ion flame detector was utilized, with a detection temperature also maintained at 180 °C. The carrier gas used was nitrogen with a pressure of 60 kPa, while the hydrogen and oxygen pressures were set at 50 kPa each. The sensitivity level was adjusted to 101, and the attenuation was set to 3.0.

### 2.8. Bacterial DNA Extraction and 16S rDNA Gene Sequencing

Microbial genomic DNA from the colonic digest was extracted using the QIAamp DNA Stool Mini Kit (Qiagen, Hilden, Germany). The V3–V4 region of the bacterial 16S rDNA was amplified with primers 341F (5′-CCTAYGGGRBGCASCAG-3′) and 806R (5′-GGACTACHVGGGTWTCTAAT-3′) using the following program: an initial denaturation at 98 °C for 2 min, followed by 30 cycles of denaturation for 10 s at 98 °C, annealing for 30 s at 50 °C, and extension for 30 s at 72 °C, concluding with a final extension at 72 °C for 8 min. The PCR products were purified using the QIAquick PCR Purification Kit (Qiagen, Hilden, Germany). The purified amplicons were then prepared utilizing the TruSeq DNA PCR-Free Library Preparation Kit for Illumina (New England Biolabs, Massachusetts, MA, USA). Sequencing was performed on the Illumina HiSeq platform (Novogene Bioinformatics Technology Co., Ltd., Beijing, China).

### 2.9. Statistical Analysis

Excel was used to organize the data. Data analysis was performed using IBM SPSS statistics 27.0 software. A one-way ANOVA was used to analyze the differences in various environmental parameters statistically. GraphPad Prism 9.5 software was used to plot the results. The Turkey test of one-way ANOVA was used to analyze its significance. When the data did not conform to the normal distribution, the Spearman correlation analysis method was used to analyze the correlation between different environmental parameters at different monitoring sites. The significance level was declared as *p* ≤ 0.05, *p* < 0.01 is extremely significant, and the results are expressed as “mean ± SEM”.

## 3. Results

### 3.1. Growth Performance, Diarrhea Score, and Organ Index

The effects of dietary supplementation with varying concentrations of MEO on the growth performance of weaned piglets are presented in Table 2. Compared with the CON group, the inclusion of MEO resulted in an increase in both ADG and ADFI among the weaned piglets, however, only the ADG of the L-MEO group showed a significant increase (*p* < 0.05), while the other treatment groups did not demonstrate statistically significant differences. Additionally, the addition of MEO to the diet can reduce FCR, of which the FCR of the L-MEO group and H-MEO group FCR are significantly reduced. However, the difference between the M-MEO group is insignificant. The inclusion of MEO also significantly decreased the diarrhea rate (*p* < 0.01), of which the M-MEO effect was the best. The organ index is shown in Table 2, and there was no statistical significance in the heart, spleen, pancreas, or lungs, or any other indicators. Compared with the CON group, the liver index of both the M-MEO group and H-MEO group increased significantly, and there was no significant difference between the L-MEO group. Additionally, the kidney index in the H-MEO group was significantly higher than that in the other MEO group, although there was no significant difference when compared to the control group.

### 3.2. Intestinal Tissue Morphology and Structure

**Figure 1:**
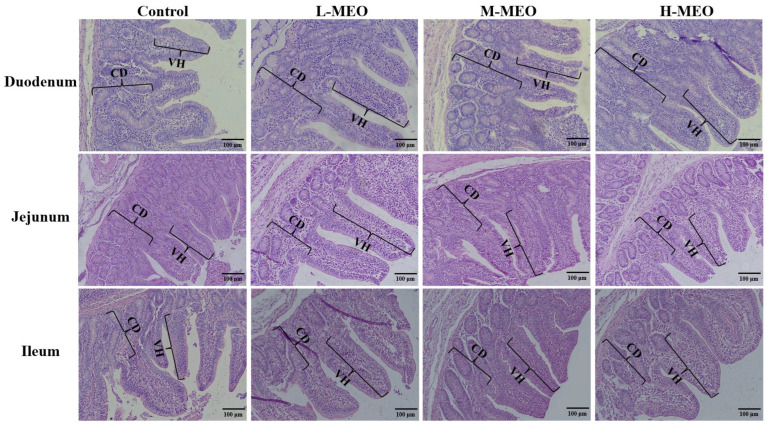
Effects of different concentrations of MEO on the intestinal morphology in weaned piglets. The hematoxylin and eosin-stained intestinal cross-sections in piglets are shown, with a scale bar representing 100 μm. CD: crypt depth; VH: villi height. CON: basal diet; L-LEO: basal diet supplemented with 300 mg/kg MEO; M-LEO: basal diet supplemented with 500 mg/kg MEO; H-LEO: basal diet supplemented with 700 mg/kg MEO; MEO: microencapsulated essential oils.

Intestinal morphology was examined using H&E staining in the weaned piglets (Figure 1). The effects of varying concentrations of MEO on the intestinal tissue morphology and structure of weaned piglets are detailed in Table 3. Compared with the CON group, dietary supplementation with MEO significantly increased the length of the jejunum and the total length of the small intestine, among which the L-MEO group and M-MEO group are both significant (*p* < 0.05). In contrast, the H-MEO group exhibited only an increasing trend. Additionally, there was no statistical significance in the duodenum and ileum.

The effects of adding different concentrations of MEO supplementation in the diet on the villus height, crypt depth, and V:C ratio in the duodenum, jejunum, and ileum of piglets are presented in Table 3. Compared with the CON group, adding MEO can significantly increase the V:C ratio of the duodenum of weaned piglets (*p* < 0.01). In weaned piglets, the height of the jejunal villi in the L-MEO group and M-MEO group were significantly greater than in the CON group (*p* < 0.05). In contrast, the height of the H-MEO group increased but not significantly. Feeding MEO can reduce the depth of crypts in the duodenum and jejunum of piglets. Dietary supplementation with MEO significantly increased the height of ileum villi in piglets, while the H-MEO group increased the depth of crypts. Except for the H-MEO group, both the L-MEO group and the M-MEO group V:C ratio increased significantly (*p* < 0.05). The above results show that adding an MEO supplement can effectively improve the intestinal morphology of weaned piglets.

### 3.3. Intestinal Antioxidant Capacity

The results of the effects of adding different concentrations of MEO to the diet on the intestinal antioxidant capacity of weaned piglets are shown in Table 4. The results showed that the addition of MEO significantly increased the T-AOC activity in the jejunum of piglets (*p* < 0.05), but only L-MEO decreased the MDA concentration (*p* < 0.001). Compared with the CON group, the feeding with MEO significantly increased the ileum CAT activity (*p* < 0.05), and the MDA level decreased (*p* < 0.05). Diet treatment did not affect the antioxidant capacity of the duodenum.

### 3.4. SCFA Concentrations in Colonic Content

The effects of feeding different concentrations of MEO on the SCFAs in the colon of weaning piglets are in Table 5. It can be seen from the results that the H-MEO group significantly increased the content of isobutyric acid (*p* < 0.05) in the piglet colon. However, it did not affect the content of acetic acid, propionic acid, butyric acid, propionic acid, or isovaleric acid.

### 3.5. The Composition of Colonic Microbiota

**Figure 2:**
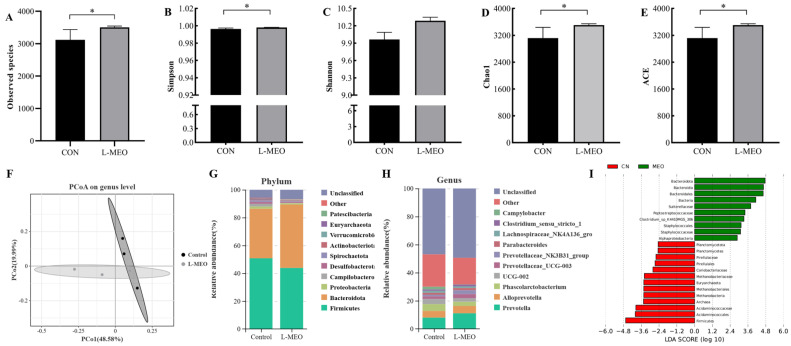
Effects of different concentrations of MEO on the diet and composition of colonic microbiota in weaned piglets. (**A**) Observed species of colonic microbiota; (**B**) Simpson index of colonic microbiota; (**C**) Shannon index of colonic microbiota; (**D**) Chao 1 index of colonic microbiota; (**E**) ACE index of colonic microbiota; (**F**) PCoA on genus-level colonic microbiota; (**G**) relative abundance on phylum level between CON and L-MEO; (**H**) relative abundance on genus level between CON and L-MEO; (**I**) shows species with significant differences in abundance among different groups. CON: basal diet; L-LEO: basal diet supplemented with 300 mg/kg MEO; MEO: microencapsulated essential oils. * *p*-value < 0.05.

The L-MEO group had the best effect on growth performance, diarrhea improvement, and intestinal health of piglets, so we selected the colonic contents for 16sRNA sequencing to investigate the effect of MEO on the abundance of intestinal microorganisms in the weaned piglets. The results showed the alpha diversity analysis of the two groups, including Sob, Simpson, Shannon, Chao, and ACE indices, which were significantly different (*p* < 0.05) except for the Shannon index, which was not different (*p* > 0.05; Figure 2A–E). Based on PCoA the confidence ellipse of the L-MEO flora deviated significantly from that of the CON (*p* < 0.05), and this index showed that there were differences in the microbial populations of the two groups of test piglets (Figure 2F). 

The colonic microbial phylum levels are presented in Figure 2G. The dominant microbiota in the colons of both the L-MEO group and CON group included Firmicutes, Bacteroidota, Proteobacteria, and Campilobacterota. In the L-MEO group, there was an increase in the mean relative abundance of Bacteroidota, Actinobacteriota, and Verrucomicrobiota, while the relative abundance of Firmicutes, Proteobacteria, and Campylobacterota was observed to decrease compared to the CON group (Figure 2G). Additionally, the relative abundances of *Prevotellaceae_UCG-003*, *Prevotellaceae_NK3B31_group*, *Parabacteroides*, and *Clostridium*_sensu_stricto_1 in the L-MEO group were higher than those in the CON group, although these differences were not statistically significant. Conversely, the proportions of *Phascolarctobacterium*, UCG-002, *Lachnospiraceae*_NK4A136_group, and *Campylobacter* were reduced, with the decreases in UCG-002 and Campylobacter reaching statistical significance (*p* < 0.05).

## 4. Discussion 

During the weaning stage, piglets are often prone to symptoms such as growth lag and frequent diarrhea due to various factors [26]. Various Studies have demonstrated that supplementation with EOs can enhance production performance and promote gut health. Su et al. [27] showed that supplementing piglet diets with 200 mg/kg EOs improved FCR and increased ADG. Some researchers have reported that similar structures incorporating EOs in piglet diets enhanced nutrient utilization and promoted gut health [28,29]. Our results showed that dietary supplementation with MEO significantly increased the ADG and ADFI of piglets while markedly reducing the FCR. This aligns with the results of previous studies [30,31]. In our results, L-MEO exhibited the most pronounced effects, which indicates that MEO does not function solely as an energy supplement. Instead, it may enhance the palatability of the feed, thereby increasing ADG and ADFI. Furthermore, the diarrhea rate has been used as a representative index of gut health [32]. It is reported that giving cinnamaldehyde (100 mg/kg) to piglets to replace antibiotics can reduce the diarrhea rate of piglets [33]. In this study, the piglets supplemented with MEO exhibited a lower diarrhea rate compared to the CON group, irrespective of dose.

The intestine as the largest immune and digestive organ in piglets, and the normal development of its morphological structure, is the physiological basis for piglets to resist pathogens and efficiently utilize nutrients [34]. In this study, the supplementation of 300 and 500 mg/kg of MEO in the diet significantly improved the jejunum and small intestine length, and there was a tendency to increase the length of the duodenum and ileum. These results indicate that MEO positively influences intestinal morphology. The height of intestinal villi, crypt depth, and the V:C ratio are fundamental factors influencing intestinal function and absorption capacity [35,36]. The intestinal villus atrophied as the epithelium decreased, and the crypts deepened as cell differentiation decreased [37]. Studies have indicated that supplementing the weaning pigment diet with thymol and cinnamaldehyde significantly increases the height of the ileum villus and the ileum V:C ratio [38]. In this study, dietary supplementation with MEO notably increased the V:C ratio in the duodenum and the villus height in the ileum. Specifically, L-MEO and M-MEO significantly enhanced villus height in the jejunum and the V:C ratio in the ileum, whereas H-MEO did not show a significant effect. This shows that one of the functions of MEO is to improve intestinal morphology, allowing nutrients to remain in the gastrointestinal tract longer, thereby enhancing nutrient absorption and improving the FCR.

In swine production, oxidative stress is a common problem and seriously impacts animal profitability [39]. Research has shown that the concentration of inflammatory cytokine after weaning piglets is raised, which can easily cause intestinal damage [40]. Most EOs are known for their antioxidation ability [41,42,43]. This experiment showed that adding MEO to the diet increased T-AOC and CAT activities in the duodenum, T-SOD and CAT activities in the jejunum, and T-SOD activity in the cecum. The above results indicate that MEO can enhance intestinal antioxidant capacity. In addition, the MDA levels in the jejunum of the H-MEO were significantly higher than in the other three groups, which indicates that it may cause piglet intestinal oxidation stress. The L-MEO is the best concentration for the cost of MEO, its effect on piglet production performance, and improving intestinal health. Therefore, we focused our 16S rRNA testing on CON and L-MEO.

SCFAs is the main metabolite of microorganisms in the cecum and colon of animals, which mainly include acetic acid, propionic acid, and butyric acid [44]. It can promote intestinal epithelial cell proliferation and mucosal growth, and is important for maintaining intestinal stability and promoting intestinal health [45]. Previous studies have found that supplementing the diet with EOs can significantly increase the concentration of SCFAs in the intestine of pigs [43,46]. The experimental results show that dietary supplementation with MEO significantly increased isobutyric acid concentration, and acetic acid concentration showed an upward trend. Branched-chain fatty acids mainly reflect protein digestion. The increase in isobutyric acid concentration in the cecum after feeding with MEO may be due to the increased digestibility of crude protein, but intestinal microorganisms mainly drive specific changes. 

The intestinal microbiota is essential for maintaining intestinal homeostasis and animal health [47]. It can participate in the absorption and metabolism of the host’s nutrients, regulate the body’s metabolism, and maintain the body’s immune function [48,49]. In our research, we sequenced and analyzed the cecal digesta, which reflects the species richness of gut microbiota. This paper used the Shannon, ACE, Sobs, and Chao indices as qualitative alpha diversity measures [20,50]. In the present study, MEO supplementation enhanced the Shannon and Chao 1 indices of the cecal microbiota. To further explore variations in how MEO affects pathogenic and beneficial bacteria at the genus level in the cecal, the structure of gut microbiota was analyzed. The level of intestinal flora in this study, the relative abundance of *Bacteroidota* and *Actinobacteriota* was increased, and the relative abundance of Firmicutes Proteobacteria was decreased. Clinical studies have also found significant differences in the gut microbiota between healthy and obese patients, especially in the abundance of Firmicutes and *Bacteroidota* [51]. Studies have shown that the ratio of Firmicutes to Bacteroidetes is associated with obesity, and an increase in the abundance of Bacteroidetes will be beneficial for controlling obesity [52]. This may not be a good indicator in swine production. In our results, MEO supplementation decreased the abundance of Firmicutes and increased the abundance of Bacteroidetes, but fortunately, it does not affect the feed-to-weight ratio of piglets. As a core component of one of the two most common bacterial enterotypes in swine gut microbiota, *Prevotella*-driven enterotypes have been shown to correlate positively with animal performance, including feed intake, feed effectiveness, and weight gain. They can also increase fat accumulation in pigs [53,54]. *Alloprevotella* can ferment carbohydrates to produce short-chain fatty acids, mainly acetic and succinic acids [55,56]. In the colon, dietary MEO supplementation markedly increased the relative abundance of beneficial bacteria, indicating that MEO encouraged the proliferation of probiotics. We found that the abundance of *Phascolarctobacterium*, UCG-002, and *Parabacteroides* in the Firmicutes phylum decreased relatively, which may be because supplementing the diet with MEO made the feed easier to digest. Studies have shown that protein restriction in pigs is associated with decreased lactobacilli, streptococci, and SCFA levels, and higher levels of *Prevotella* [57]. The results from the intestinal microbiota analysis showed that adding MEO can significantly adjust the intraunion to degrade crude fiber protein and bring polysaccharides into the diets.

## 5. Conclusions

This study demonstrated that dietary MEO can enhance growth performance and intestinal health in weaned piglets. The beneficial effects may be attributed to improvements in intestinal morphology, increased antioxidant capacity, and modulation of microbial communities. The results provide a theoretical foundation for the application of carvacrol and cinnamaldehyde as natural plant extracts to promote intestinal health and appetite for weaned piglets. In addition, the inclusion of 700 mg/kg MEO may diminish the intestinal absorption area and aggravate intestinal oxidative stress in piglets, whereas supplementation with MEO at the recommended dosage of 300 mg/kg was found to be advantageous.

## Figures and Tables

**Table 1:** Ingredient composition and nutritional levels of basic diet.

Items	Content	Items	Content
Composition of ingredients		Nutrient levels ^2^	
Corn	59	Net energy/(MJ·kg^−1^) ^3^	11.07
Soybean meal	6	Crude protein	17.59
Extruded soybean	14	Crude fat	6.63
Soybean oil	1.5	Crude ash	5.97
Dried whey	5	Total calcium	0.55
Fermented soybean meal	4	Total phosphorus	0.55
Montmorillonite	0.5	Digestible lysine	1.22
Premix ^1^	10		
Total	100		

^1^ The premix feed provides the following nutritional components per kilogram of feed: copper sulfate 10 mg, ferrous sulfate 80 mg, manganese sulfate 80 mg, zinc sulfate 75 mg, potassium iodide 0.40 mg, sodium selenite 0.30 mg, vitamin A 8000 IU, vitamin B1 4 mg, vitamin B2 3.6 mg, vitamin B5 40 mg, vitamin B6 4 mg, vitamin B12 0.02 mg, vitamin D3 3000 IU, vitamin E 20 IU, vitamin K3 2 mg, biotin 0.15 mg, folic acid 1.0 mg, d-pantothenic acid 11 mg, niacin 10 mg, and antioxidant 100 mg. ^2^ Nutrient levels were calculated according to NRC (2012). ^3^ Net Energy was a calculated value, the rest were measured values.

**Table 2:** Effect of different concentrations of MEO on growth performance, diarrhea score, and organ index in weaned piglets.

Item	Treatments ^1^				SEM ^3^	*p*-Value
	CON	L-MEO	M-MEO	H-MEO		
ADG(g/d) ^2^	212.02 ^b^	281.25 ^a^	254.4 ^ab^	256.55 ^ab^	10.28	0.045
ADFI(g/d) ^2^	383.65	431.12	402.73	403.17	9.26	0.120
FCR^2^	1.81 ^a^	1.54 ^b^	1.61 ^ab^	1.57 ^b^	0.04	0.037
Diarrhea scores (%)	26.36 ^a^	14.03 ^b^	10^b^	11.52 ^b^	2.23	0.001
Organ index (g/kg)						
Heart index	5.04	5.05	5.08	5.21	0.12	0.677
Liver index	22.31 ^b^	22.33 ^b^	25.65 ^a^	25.71 ^a^	0.58	0.023
Spleen index	1.76	1.77	1.75	2.22	0.09	0.100
Kidneys index	5.59 ^ab^	5.27 ^ab^	5.2 ^b^	6.93 ^a^	0.3	0.018
Pancreas index	2.62	2.5	2.52	2.82	0.1	0.335
Lungs index	15.37	14.32	17.92	15.19	1.21	0.395

^a,b^ Means in the same row with different superscripts are significantly different (*p* < 0.05). ^1^ CON: basal diet; L-LEO: basal diet supplemented with 300 mg/kg MEO; M-LEO: basal diet supplemented with 500 mg/kg MEO; H-LEO: basal diet supplemented with 700 mg/kg MEO; MEO: microencapsulated essential oils. ^2^ ADG: average daily gain; ADFI: average daily feed intake; FCR: average daily feed intake/average daily gain. ^3^ SEM, standard error of the mean (*n* = 3).

**Table 3:** Effect of different concentrations of MEO on intestinal tissue morphology and structure in weaned piglets.

Item	Treatments ^1^				SEM ^2^	*p*-Value
	CON	L-MEO	M-MEO	H-MEO		
Intestine length, cm						
Duodenum length	35.33	37.77	37.63	36.83	2.7	0.803
Jejunum length	310.1 ^b^	397.53 ^a^	368.2 ^a^	350.57 ^ab^	11.73	0.027
Ileum length	420.7	480.93	478.8	437.73	11.15	0.063
Small intestine length	766.13 ^b^	916.23 ^a^	884.63 ^a^	825.13 ^ab^	22.46	0.032
Villus height, μm						
Duodenum	322.16	346.23	335.64	322.57	5.05	0.130
Jejunum	310.21 ^b^	367.1 ^a^	369.9 ^a^	329.54 ^ab^	9.6	0.039
Ileum	268.14 ^b^	375.33 ^a^	331.64 ^a^	349.37 ^a^	14.06	0.013
Crypt depth, μm						
Duodenum	254.16	228.88	223.79	227.32	5.72	0.088
Jejunum	231.34	197.24	224.04	218.81	7.98	0.205
Ileum	198.75	202.46	186.93	239.66	9.09	0.062
Villi height/Crypt depth						
Duodenum	1.27 ^b^	1.52 ^a^	1.50 ^a^	1.42 ^a^	0.03	0.006
Jejunum	1.35 ^b^	1.86 ^a^	1.68 ^ab^	1.53 ^sb^	0.08	0.047
Ileum	1.35 ^b^	1.86 ^a^	1.79 ^ab^	1.48 ^ab^	0.08	0.033

^a,b^ Means in the same row with different superscripts are significantly different (*p* < 0.05). ^1^ CON: basal diet; L-LEO: basal diet supplemented with 300 mg/kg MEO; M-LEO: basal diet supplemented with 500 mg/kg MEO; H-LEO: basal diet supplemented with 700 mg/kg MEO; MEO: microencapsulated essential oils. ^2^ SEM, standard error of the mean (*n* = 3).

**Table 4:** Effect of different concentrations of MEO on intestinal antioxidant capacity in weaned piglets.

Item	Treatments ^1^				SEM ^3^	*p*-Value
	CON	L-MEO	M-MEO	H-MEO		
Duodenum						
T-SOD (U/mg prot) ^2^	67.22	59.32	66.22	64.03	2.68	0.398
MDA (nmol/mg prot) ^2^	2.13	0.91	2.02	2.18	0.27	0.146
T-AOC (nmol/mg prot) ^2^	0.07	0.07	0.08	0.08	0.003	0.078
CAT (U/mg prot) ^2^	2.38	3.02	2.97	3.18	0.16	0.115
Jejunum						
T-SOD (U/mg prot)	37.12	48.71	44.83	44.94	2.09	0.083
MDA (nmol/mg prot)	1.69 ^b^	0.74 ^c^	1.72 ^b^	4.4 ^a^	0.43	*p* < 0.001
T-AOC (nmol/mg prot)	0.035 ^b^	0.058 ^a^	0.049 ^ab^	0.044 ^ab^	0.003	0.011
CAT (U/mg prot)	1.95	3.12	2.9	2.93	0.27	0.198
Ileum						
T-SOD (U/mg prot)	54.14	69.04	58.52	58.94	3.15	0.154
MDA (nmol/mg prot)	2.92 ^a^	2.56 ^ab^	1.8 ^b^	2.10 ^ab^	0.19	0.039
T-AOC (nmol/mg prot)	0.037 ^b^	0.083 ^a^	0.06 ^ab^	0.055 ^ab^	0.007	0.015
CAT (U/mg prot)	1.91 ^b^	2.91 ^a^	2.67 ^a^	2.66 ^a^	0.14	0.033

^a,b,c^ Means in the same row with different superscripts are significantly different (*p* < 0.05). ^1^ CON: basal diet; L-LEO: basal diet supplemented with 300 mg/kg MEO; M-LEO: basal diet supplemented with 500 mg/kg MEO; H-LEO: basal diet supplemented with 700 mg/kg MEO; MEO: microencapsulated essential oils. ^2^ T-SOD: total superoxide dismutase; MDA: malondialdehyde; T-AOC: total antioxidant capacity; CAT: catalase. ^3^ SEM, standard error of the mean (*n* = 3).

**Table 5:** Effect of different concentrations of MEO on SCFAs in weaned piglets.

Item	Treatments ^1^				SEM ^2^	*p*-Value
	Con	L-MEO	M-MEO	H-MEO		
Acetic acid, mg/g	0.255	0.262	0.288	0.319	0.019	0.333
Propionic acid, mg/g	0.185	0.153	0.179	0.234	0.015	0.098
Butyric acid, mg/g	0.075	0.062	0.062	0.095	0.009	0.279
Isobutyric acid, mg/g	0.047 ^b^	0.069 ^ab^	0.086 ^ab^	0.116 ^a^	0.01	0.012
Valeric acid, mg/g	0.013	0.011	0.013	0.019	0.01	0.184
Isovaleric acid, mg/g	0.077	0.055	0.068	0.090	0.007	0.096
Total SCFAs, mg/g	0.651	0.612	0.696	0.872	0.055	0.148

^a,b^ Means in the same row with different superscripts are significantly different (*p* < 0.05). ^1^ CON: basal diet; L-LEO: basal diet supplemented with 300 mg/kg MEO; M-LEO: basal diet supplemented with 500 mg/kg MEO; H-LEO: basal diet supplemented with 700 mg/kg MEO; MEO: microencapsulated essential oils. ^2^ SEM, standard error of the mean (*n* = 3).

## Data Availability

The original contributions presented in the study are included in the article/supplementary material, further inquiries can be directed to the corresponding author/s.
